# Neuro-fuzzy based model of batch fermentation of *Kluyveromyces marxianus* var. *lactis* MC5

**DOI:** 10.1080/13102818.2014.944364

**Published:** 2014-10-30

**Authors:** Tatiana Ilkova, Mitko Petrov

**Affiliations:** ^a^ Bulgarian Academy of Sciences, Institute of Biophysics and Biomedical Engineering, Sofia, Bulgaria

**Keywords:** neural network, neuro-fuzzy neural network, fuzzy sets theory, *Kluyveromyces marxianus* var. *lactis* MC5

## Abstract

In this work a neuro-fuzzy based model of a whey batch fermentation process by a strain *Kluyveromyces marxianus* var. *lactis* MC5 is presented. A three-layered neuro-fuzzy network is realized. The simulation results are compared with conventional models (based on mass balance and differential equations). The neuro-fuzzy model provides a better fitness and allows inclusion of linguistic variables (such as colour, smell, taste, morphophysiology, etc.). The accuracy is approximately equal to this achieved by a conventional neural network. The proposed approach is flexible (with regard to the process model) and quite robust (with regard to the possible uncertainties and to the optimization surface). Future work will focus on applying this approach for modelling of different biotechnological processes.

## Introduction

The fermentation of lactose from natural substrate by *Kluyveromyces marxianus* var. *lactis* MC5 is a non-conventional way of obtaining unicellular protein. This process is not well studied due to the extreme complexity and variety of microbial metabolic activities. That is why, there does not exist a general mathematical model of the microbial biosynthetic process, although there are various models of biotechnological processes and of different parts of the whey fermentation. Cheese whey, which is a waste product from the production of white brine cheese, can be utilized, thus allowing for the production cycle to be closed. The conventional model includes the dependence between the concentrations of the basic factors measured during the fermentation process: lactose, oxygen and cell mass.[1]

Neural networks (NNs) can be considered as universal approximations. This property of NN is used in their application for modelling the dynamics of biotechnological processes.[2,3,4–8] Interesting and promising algorithms for training NNs have been proposed by using paradigms from the fuzzy set theory. The main advantage of neuro-fuzzy networks (NFNs) as a ‘flexible’ model is that they allow modelling of complex and ill-defined objects. However, the learning algorithms commonly used (backpropagation, reinforcement learning, etc.) are very time consuming.[9,10,11–13]

A simplified type of NFN is considered in this study. This NFN consists of three layers. The transfer functions of every neuron at the second layer (from hidden neurons to the output neuron) are considered to be piece-wise linear. A powerful tool for more ‘flexible’ description that can be considered as more appropriate and closer to the biological nature of the neuronal action are fuzzy functions.[5,6,10,13]

Therefore, NNs are considered as an alternative technique which is very effective in cases of complex and sophisticated plants.

The aim of this study was to develop a neuro-fuzzy model of a batch cultivation of *Kluyveromyces marxianus* var. *lactis* MC5 for lactose oxidation in a natural source.

## Materials and methods

### Cultivation procedure

Six aerobic batch cultivations were carried out in a lab-scale bioreactor ABR 02M with a 2 L volume. *Kluyveromyces marxianus* var. *lactis* MC5 was cultivated in basic nutrient medium (whey ultra-filtrate with lactose concentration of 44 g/L, 0.6% (NH)HPO, 5.0 % yeast's autolisate, 1.0 % yeast extract, pH 5.0–5.2).[1] The ultra-filtrate was derived from whey from the production of white cheese, following deproteinization by ultra-filtration on LAB 38 DDS with a membrane of the GR 61 PP type under the following conditions: *T* = 40–43 °C, input pressure *P*_in_ = 0.65 MPa and output pressure *P*_out_ = 0.60 MPa. The ultra-filtrate was used in a native condition.

The velocity of the air flow was 1 L gas per 1 L broth up to the fourth hour and 2 L gas per 1 L broth up to the end of the process under continuous mixing at 800 r/min). The temperature was 29 °C. The cultivation lasted 12 h (final time, *t_f_* = 12 h).

### Analytical measurements

The dynamics of the microbiological process (lactose conversion to protein in yeast cells) were studied during the strain growth. The lactose concentration in the fermentation medium in oxidation and assimilation of lactose by *K. lactis* was determined by enzyme methods by UV tests. The concentration of cell mass and the protein content were determined on the basis of Kjeldahl nitrogen analysis (Kjeltec 1028 Analyzer). The concentration of the dissolved oxygen in the fermentation medium in the process of oxidation and assimilation of lactose was determined by an oxygen sensor.

The dynamics of the biotechnological processes can be described by differential equations which represent mutual dependences of cell mass (*X*), substrate (*S*), dissolved oxygen concentration (*C*), product (*P*), temperature (*T*), pH, etc. Discretization is usually possible due to the slow changes in these processes.

### Neuro-fuzzy based model

The model of the batch fermentation processes of the lactose oxidation by *K. marxianus* var. *lactis* MC 5 includes the dependence between the concentrations of the basic parameters: cell mass (*X*), lactose (*S*) and oxygen (*C*):(1) 
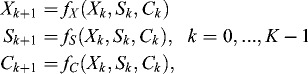
where *K* is the number of time partitions, and *f_X_*, *f_S_*, *f_C_* are non-linear functions.

Equation (1) is only a theoretical assumption. NFN is used for approximation of the non-linear functions *f_X_*, *f_S_*, *f_C_* by learning on real data. The following three-layered NFN based model is considered, in which five neurons are used in the middle layer.

**Figure 1.**
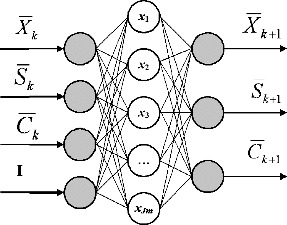
Structure of neuro-fuzzy neural network.

The structure of the proposed NFN is shown in Figure 1.

Vector 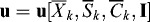 is a normalization vector of the input signals, *i* = 1, …, *INP*, where *INP* is the number of input signals; **I** is a single vector, with size **I** = 1. Vector 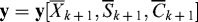 is a normalization vector of the output signals, *j* = 1, …, *OUT*, where *OUT* is the number of output signals. The vectors **u** and **B** are normalized values which belong to the interval [0, +1].

Therefore, NN is considered as an alternative technique which is very effective in cases of complex and sophisticated organisms.

**Figure 2.**
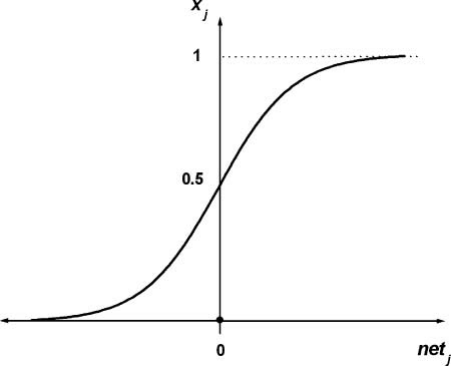
Sigmoidal function for first layer.

The transfer function at the first layer is sigmoid (Figure 2):(2) 
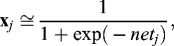

where **x** *_j_, j =* 1, …, *J_m_* is the number of hidden signals; 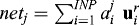, 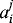 is a matrix with random weights to the interval [0, +1], *i* = 1,…, *INP*; *r* = 2, …, *R* is the size of the training set.

**Figure 3.**
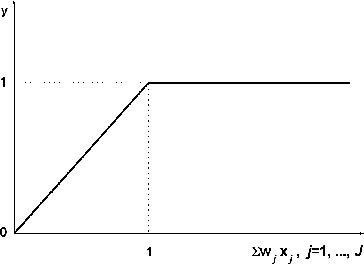
Piece-wise second layer of NFN.

The somatic mapping at the second layer is represented as a piece-wise linear function [5,14] based on a fuzzy equation (Figure 3):(3) 
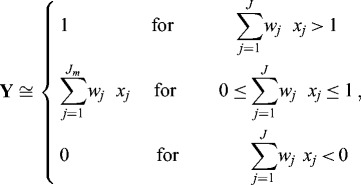

where ‘≅’ is a fuzzy equation represented by its membership function, and *wj* are the weights of the hidden neurons.

The least-squares error is a commonly used criterion for the accuracy of the computed profiles of the state variables as compared to the experimental measurements. The training task includes determination of the time-weighted least-squares error as a criterion for each experiment. The criterion is expressed in the following form [15]:(4) 
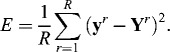



The membership function of the criterion (Equation (4)) is given as follows:(5) 
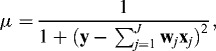
where **y** is a vector of the experimental data, 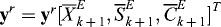; 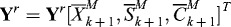 is a vector from the training set that denotes the fuzzy minimization.

Generally, the training task is represented as follows:(6) 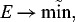

(7) 
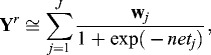
where ‘
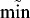
’ denotes fuzzy minimization.

This problem is of the class of the fuzzy mathematical programming problems.[1,9,14] As a result, the best possible weights will be found as a solution of the following system of linear equations:(8) 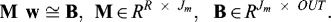



The elements of the matrices **M** and **B** are determined by the following dependences:(9) 
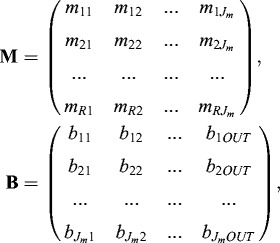
where *r* = 2, …, *R*; 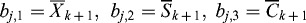, *j* = 1, …, *J_m_*,


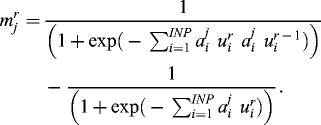


The best possible weights of the neurons in the second layer are considered after solving Equation (8). As far as the number of neurons in the first layer is known (equal to the number of the input signals), the number of neurons is subject to determination. It is determined simultaneously with the training of the NN.

The fuzzy equalities are used in order to represent more adequate somatic mapping. Therefore, the training task is a fuzzy optimization problem. By applying the results in this field [9,14] and its possibilities for NNs learning by a non-iterative algorithm for analytical training, the above-mentioned type of an NFN is proposed in this work. This type of NFN is proposed as a simple non-iterative algorithm for training:

### Training algorithm

BEGIN

1. Input the initial data: 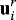, *
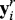
* *INP, OUT* *, J_m_* and *K*. In this case: *INP =* 4, *OUT* = 3,*J_m_* = 5, *K* = 13 and *R* = 6.
2. By randomization, determine the coefficients 
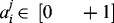
,  *j* = 1, …, *J_m_*, *i* = 1, …, *INP*.
3. Find the maximal values of input (**u**_max_) and output signals (**y**_max_).
4. Normalize vectors **u** and **y** in interval [0, +1] by equations 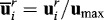 and 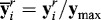.
5. Compute the elements of the matrices **M** and **B** from Equation (9).
6. Solve the linear system equation **M w ≅ B** with subroutine LIN_SOL_SVD from IMSL MP 90 library of COMPAQ Visual FORTRAN 90 Pro.
7. Simulate the results with neuro-fuzzy based model.
8. Print results: *INP* – number of input signals, *OUT* – number of output signals, *J_m_* – number of hidden neurons in second layer, *R* – number of data in training sets, **w** – weights vector, **w** = **w**[**w** *_X_* **w** *_S_* **w** *_C_*]. Print the simulation and predict with NFN with real data:
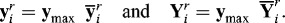

END

The developed programs were written using the COMPAQ Visual FORTRAN 90 Pro language. All computations were performed on an AMD Athlon II X2 245, 2.9 GHz computer using Windows XP operating system.

## Results and discussion

The training of the NN was done on the basis of the developed algorithm and programme. After the training of the NFN, the next values for the number of the neurons in the second layer and their weights were obtained, *J* = 5:


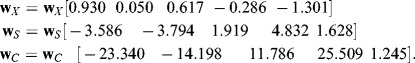


### Comparative analysis between conventional and neuro-fuzzy model

The best dependences are defined by the statistical criteria: experimental correlation quotient 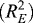, experimental Fisher function (*F_E_*), relative error (*S_L_*), and statistic λ for conventional and neuro-fuzzy models (NFM) for output kinetic variables (*X_k_*_+1_), (*S_k_*_+1_) and (*C_k_*_+1_).[16]

A statistical analysis of the values predicted with conventional and neuro-fuzzy models was made (Table 1). The theoretical function of Fisher was *F_T_*(3, 12) = 3.49. The theoretical function of Fisher for statistic λ was found to be *F^′^_T_*(3, 6) = 4.76, and the theoretical correlation coefficient was obtained to be *R* ^2^ *_T_*(10) = 0.516.[17]

**Table 1.** Statistical results of conventional and neuro-fuzzy based model.

			*F_E_*	
Variables	Conventional model	Neuro-fuzzy model	Conventional model	Neuro-fuzzy model
*X*	0.9905	1.0000	0.9385	0.9245
*S*	0.9882	0.9987	0.9031	0.8906
*C*	0.9980	0.9985	1.0411	0.9783
				
	*S_L_*		*Statistic *λ	
Variables	Conventional model	Neuro-fuzzy model	Conventional model	Neuro-fuzzy model
*X*	0.5218	0.0322		
*S*	5.0466	0.1902	1297.4	6018.6
*C*	0.4570	0.2389		

The results in Table 1 show that the neuro-fuzzy model has better indexes than the conventional model, accordingly it has a larger correlation coefficient 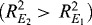, a lower Fisher transformation value 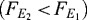, a lower relative error 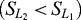 and, last but not least, a higher value for the statistic λ_2_ *≫ *λ_1_. This was true for all kinetic variables of the examined process.

**Figure 4.**
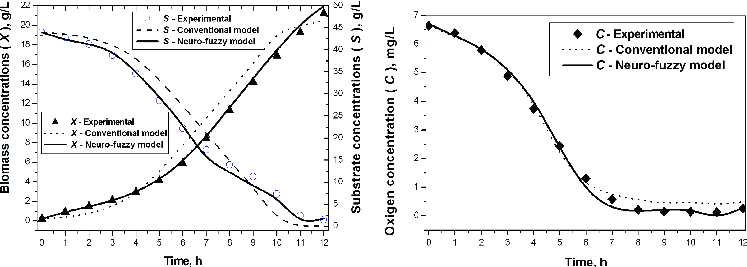
Experimental and simulation results with a conventional and neuro-fuzzy model. Simulation of biomass and substrate concentration (a) and simulation of oxygen concentration (b).

The results after simulations with the conventional and the neuro-fuzzy based model for kinetic variables are shown in Figure 4(a) and 4(b).

The obtained results (Table 1, Figure 4) show that the neuro-fuzzy model very well describes the experimental data. This approach allows training on NFN for short time (∼3 min). The accuracy is approximately equal to this achieved by a conventional NN.

These promising results indicate that utilization of the NN is advisable for description of processes as complex as biotechnological ones. With this the necessity for the solution of a system of ordinary differential equations and subsequent parameter identification, which would require more experimental data than those necessary for training of the NN. Further research will be needed to apply this approach for modelling of different biotechnological processes.

## Conclusions

A conventional and a neuro-fuzzy neural model of a batch whey fermentation process are compared as a result of a study on a microbial process. The neuro-fuzzy neural model has flexible structure and linguistic variables can be used. This approach allows training a neuro-fuzzy model for a short time (∼3 min). The accuracy is approximately equal to this achieved by conventional methods. The utilization of the NN is advisable for description of such complex processes as biotechnological ones. With this the necessity for solving a system of ordinary differential equations and subsequent parameter identification is circumvented, reducing the amount of experimental data needed. Further research will explore the potential application of this approach for modelling of different biotechnological processes.

## Funding

This work was supported by Operative Programme ‘Human Resources Development’ [grant number BG051PO001-3.3-05-0001], ‘Science-Business’ scheme.
